# Simulation and Identification of the Habitat of Antarctic Krill Based on Vessel Position Data and Integrated Species Distribution Model: A Case Study of Pumping-Suction Beam Trawl Fishing Vessels

**DOI:** 10.3390/ani15111557

**Published:** 2025-05-27

**Authors:** Heng Zhang, Yuyan Sun, Hanji Zhu, Delong Xiang, Jianhua Wang, Famou Zhang, Sisi Huang, Yang Li

**Affiliations:** 1 Key Laboratory of Oceanic and Polar Fisheries, Ministry of Agriculture and Rural Affairs, East China Sea Fisheries Research Institute, Chinese Academy of Fishery Sciences, Shanghai 200090, China; zhangziqian0601@163.com (H.Z.); sunyuyan2022@163.com (Y.S.); mikey0987@163.com (H.Z.); xdl17852167218@163.com (D.X.); wjh20001231@163.com (J.W.); 13276209271@163.com (F.Z.); huangsisi3254@163.com (S.H.); 2 Laoshan Laboratory, Qingdao Marine Science and Technology Center, Qingdao 266237, China; 3 College of Navigation and Ship Engineering, Dalian Ocean University, Dalian 116023, China; 4 College of Marine Living Resource Sciences and Management, Shanghai Ocean University, Shanghai 201306, China

**Keywords:** Antarctic krill, vessel position data, environmental data, species distribution model, habitat research

## Abstract

This study, based on the vessel position data of pump-suction beam trawlers and the integrated species distribution model (ISDM), deeply analyzes the spatio-temporal distribution characteristics of the habitat of Antarctic krill and the contributions of key environmental factors. The Convolutional Neural Network–attention model (CNN–attention model) was used to identify the fishing status of the vessel position data of Norwegian pump-suction beam trawlers for Antarctic krill during the fishing seasons from 2021 to 2023. Variables of marine environment, including sea surface temperature (SST), sea surface height (SSH), chlorophyll concentration (CHL), sea ice concentration (SIC), sea surface salinity (SSS), and spatial factor Geographical Offshore Linear Distance (GLD) were combined and input into the ISDM for simulating and predicting the spatial distribution of the habitat. The model results show that the Area Under the Curve (AUC) and True Skill Statistic (TSS) indices for all months exceed 0.9, with an average AUC of 0.997 and a TSS of 0.973, indicating extremely high accuracy of the model in habitat prediction. Further analysis of environmental factors reveals that Geographical Offshore Linear Distance (GLD) and chlorophyll concentration (CHL) are the main factors affecting habitat suitability, contributing 34.9% and 25.2%, respectively, and their combined contribution exceeds 60%. In addition, factors such as sea surface height (SSH), sea surface temperature (SST), sea ice concentration (SIC), and sea surface salinity (SSS) have impacts on the habitat distribution to varying degrees, and each factor exhibits different suitability response characteristics in different seasons and sub-regions. There is no significant correlation between the habitat area of Antarctic krill and catch (*p* > 0.05), while there is a significant positive correlation between the fishing duration and the catch (*p* < 0.001), indicating that a longer fishing duration can effectively increase the Antarctic krill catch.

## Simple Summary

Antarctic krill is an important economic species, and its habitat and distribution are significantly affected by changes in marine factors such as distance from the shore, chlorophyll content, and water temperature. In this study, in order to address the specific situation where commercial fishing logs are difficult to obtain, a species-integrated distribution model was adopted to fit the position data of Antarctic krill pump-suction trawlers with marine environmental data. This was used to analyze the important environmental factors affecting the habitat of Antarctic krill. Additionally, the areas of habitats of different grades were fitted with the catch, as well as the fishing duration with the catch, to explore the factors influencing the fishing catch. Overall, this study provides a new method for accurately identifying the important environmental factors affecting the habitat of Antarctic krill. It can provide valuable decision-making references for the fishing operations of Antarctic krill fishing vessels and contribute to the sustainable development and utilization of Antarctic krill resources.

## 1. Introduction

Antarctic krill (*Euphausia superba*), as a key species in the Southern Ocean ecosystem [1], holds a unique position in the field of global biological resources. It is not only the single species with the largest resource quantity, but also contains considerable economic and ecological values that cannot be ignored. Given the close correlation between its habitat distribution and the marine environment, accurately grasping the dynamic changes in krill fishery resources has become a core and crucial element for improving fishery production efficiency [2].

The marine environment is one of the key factors affecting the habitat distribution of krill. Numerous studies have shown that environmental variables such as sea surface temperature, sea ice, and chlorophyll concentration significantly influence the spatio-temporal distribution characteristics of krill habitats [3,4,5,6]. In addition, physical spatial factors such as the distance from the shore and the sea surface height also have a significant impact on the distribution of krill [6,7]. Other environmental factors, such as salinity, also have a certain effect on the habitat of krill [8], but the significance of their effect remains unclear. Although how these marine environmental and physical spatial factors affect the habitat and fishery distribution of krill has been widely studied, most of these studies rely on fitting the fishing log data of commercial vessels with environmental factors. Since only 8 to 13 global krill vessels conduct fishing activities in the Antarctic FAO 48 fishing area every year, these fishing log data only record the information of fishing location points and have a low spatial coverage rate. Moreover, due to the confidentiality of commercial information, it is difficult for fishery log data from different countries to be shared [9]. To some extent, this limits people’s in-depth understanding of the distribution pattern of krill habitats. Currently, there are mainly two operation methods for krill fishing: the traditional midwater variable-depth trawl (referred to as the “traditional trawl” for short) and the pump-suction truss-pole trawl (referred to as the “pump-suction trawl” for short) [10]. Compared with the traditional trawl, the pump-suction trawl connects a fish pump to the cod end and continuously pumps krill to the deck processing workshop through a hose, simplifying the operation process. It can achieve the purpose of continuous fishing without the need for hauling and setting the net, increasing the fishing efficiency by more than 50%. Since 2006, Norway has taken the lead in introducing the pump-suction trawl technology for commercial fishing. Up to now, there are only five krill fishing vessels in the world that adopt this technology. As the two Chinese pump-suction trawl vessels (which first adopted the pump-suction trawl operation method in December 2022) are in the experimental stage and have only one year of fishing time, and there is still a great lack of knowledge about the operation location of the central fishing ground of the pump-suction trawl vessels, it is more reliable to use the vessel positions and operation locations of the three Norwegian pump-suction vessels to simulate and identify the distribution of krill habitats of this fishing method.

The Automatic Identification System (AIS), as a modern navigation and position monitoring device, can realize the automatic identification between vessels and the shore end or between vessels, and can accurately provide information including time, latitude and longitude, speed, course, etc., in real time [11]. Since fishing logs are difficult to obtain and belong to commercially confidential data, analyzing the behavior of fishing vessels based on AIS data, determining the position status of krill vessels, and identifying and simulating the distribution information of fishing grounds according to the position data of the fishing status have become a new approach to studying the habitats of krill. In recent years, many scholars have applied AIS data, combined with technologies such as the threshold method and deep learning, and successfully achieved high-precision identification of the fishing behavior of fishing vessels, and extracted fishery information such as the fishing locations and fishing efforts of fishing vessels [12,13,14,15]. These achievements provide a new perspective and theoretical basis for the management of fishery resources and ecological protection. Although there have been a few preliminary studies on the method for determining the position status of traditional variable-depth krill trawls and the extraction of fishing ground locations [16], there are still no relevant reports on the position status of pump-suction continuous fishing trawls and the locations of fishing grounds. This has left many research gaps in the accurate identification of the central fishing grounds of krill suitable for the pump-suction trawl fishing gear and the prediction of krill habitats, which is not conducive to the efficient fishing production of krill under the quota management system.

Currently, no relevant research has been found on analyzing the habitats of krill based on AIS vessel position data and marine environmental factors. The distribution of krill resources is uneven, and the resources of high-density fishing grounds often concentrate in areas with large marine environmental gradients and are accompanied by significant spatio-temporal changes. Marine environmental factors, such as sea temperature, sea ice, chlorophyll concentration, distance from the shore, salinity, sea surface height, etc., all have an important impact on the habitat distribution of krill [3,4,5,6]. The fishing operations of fishing vessels need to rely on the distribution characteristics of krill to determine suitable fishing areas. Usually, suitable environmental factors often mean a higher abundance of krill resources and a more obvious aggregation of krill, which also attracts fishing vessels to these areas for fishing. Since the pump-suction trawl vessels conduct continuous fishing and do not perform the operations of hauling and setting the net, their towing speed can maintain a relatively stable low speed state, and the vessels mainly aim at fishing according to the position of krill clusters, resulting in large changes in the course during the fishing process. The fishing status in the positions of pump-suction trawl vessels is an important reference basis and data source for analyzing the fishing behavior of these types of vessels and the spatio-temporal positions of krill habitats. Based on this, the fishing duration, operation hotspots, and the position distribution of krill habitats can be extracted. Therefore, in-depth research on the relationship between the fishing location points of fishing vessels and marine environmental factors helps to deeply understand the changing pattern of the abundance of krill resources and provides scientific support for the management and protection of fishery resources.

Based on the above analysis, the main research objectives of this paper are as follows: (1) Use the CNN-attention deep learning model to extract the fishing points and distribution characteristics from the AIS data of pump-suction continuous fishing vessels for Antarctic krill; (2) Fit the fishing points and environmental factors based on the integrated species distribution model (ISDM) to identify the spatio-temporal characteristics of the habitats of Antarctic krill and their environmental driving factors; (3) Explore the fitting relationship between the areas of various suitable habitat zones of Antarctic krill and the fishing characteristics (fishing duration, non-fishing duration) and the catch, laying a foundation for a deeper understanding of the relationship between the vessel position data and the distribution of the habitats of Antarctic krill resources.

## 2. Materials and Methods

### 2.1. Environmental and Fishery Data

The habitat environment of Antarctic krill is affected by environmental factors such as sea surface temperature (SST), sea surface height (SSH), chlorophyll (CHL), sea ice concentration (SIC), salinity (SSS), and distance from the shore (GLD) [6], as well as [3,4,5]. Therefore, in this paper, marine remote sensing data were downloaded from the official website of the Copernicus Marine Environment Monitoring Service (https://data.marine.copernicus.eu/ accessed on 22 May 2025) and the Global Marine Environmental Dataset (GMED—Download Data Layers). To ensure data matching, ArcGIS 10.6 was used to uniformly adjust the spatial resolution of the environmental data to 0.083° × 0.083°.

The monthly catch production data of Norwegian krill vessels during the fishing seasons from 2021 to 2023 were sourced from the Commission for the Conservation of Antarctic Marine Living Resources (CCAMLR). This dataset includes data such as the fishing country, the year and month of fishing, the fished species, and the catch production (in tons). Since the Antarctic krill fishery starts on December 1st of each year and ends in November of the same year, in this study, the fishing season of a certain year is defined as starting from December 1st of the previous year and ending on November 30th of that year. For example, the fishing season of 2021 was from 1 December 2020 to 30 November 2021.

**Figure 1:**
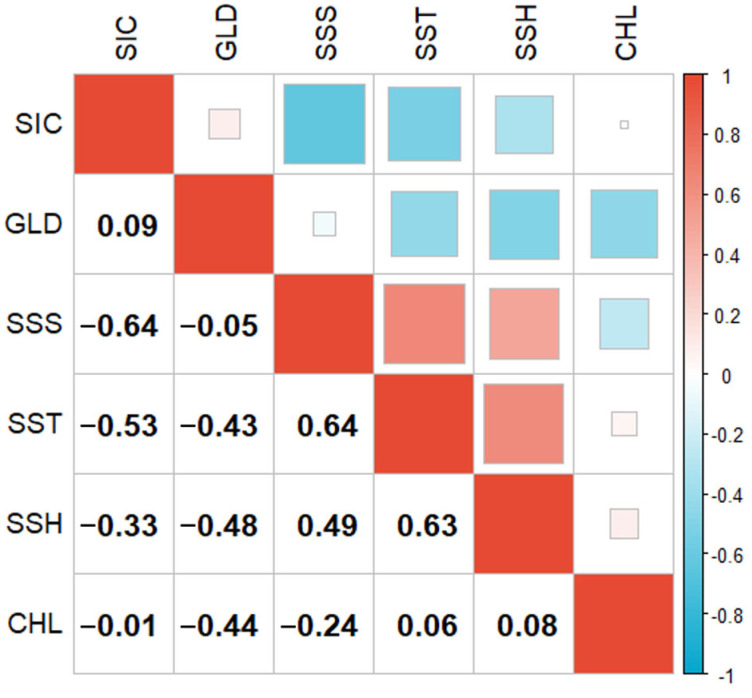
Pearson correlation test.

For the collinearity analysis of environmental factors, correlations among environmental variables are commonly present in models. Collinearity may lead to instability of parameter estimation and biases in inferential statistics [17]. By screening out environmental variables with weak correlations through correlation analysis, it is possible to optimize efficiency, reduce overfitting, and effectively improve the accuracy of model predictions. Therefore, in this paper, a Pearson correlation test was conducted on these six environmental factors (Figure 1). If the Pearson correlation coefficient is less than |0.7|, it is considered that there is no significant correlation [18]. Thus, in this study, six environmental factors, namely SIC, GLD, SSS, SST, SSH, and CHL, were selected for modeling and analysis of the factors influencing the habitat distribution of Antarctic krill.

### 2.2. Vessel Position Data

The AIS satellite constellation data source of the Canadian company exactView was ordered through an AIS commercial agent. The data include static information such as the vessel name, vessel length, gross tonnage, and Maritime Mobile Service Identity (MMSI), as well as dynamic information such as date, time, latitude and longitude, course, and speed. The fishing log data includes the name of the fishing vessel, operation time, operation location, towing speed, net mouth depth, catch amount, etc.

A total of 206,202 pieces of AIS data (excluding transshipment status data) of all Norwegian krill pump-suction trawlers (Antarctic Endurance, Saga Sea, Antarctic Sea) during the fishing seasons from 2021 to 2023 were collected. Since it is difficult to obtain the fishing logs of the Norwegian pump-suction trawlers and it is impossible to identify the location of their fishing grounds through the logs, the available AIS vessel position data and fishing logs of the same type of Antarctic krill pump-suction vessels were used to establish a vessel position identification model to determine their fishing status. Combined with environmental factors, an integrated species distribution model was used to predict and identify the habitats of Antarctic krill.

A total of 9255 pieces of AIS vessel position data of the same type of Chinese Antarctic krill pump-suction vessels during 2023–2024, and 3614 pieces of fishing log data were collected to train the vessel position status identification model (CNN–attention model). When the krill pump-suction trawler is operating, it usually sails at a high speed to the central fishing ground sea area (the vessel speed is greater than about 5 Kn and the course change is small) and then prepares for fishing (the vessel speed is greater than about 3 Kn and the course change is large). The fishing net is put into the water, the pump-suction equipment is turned on, and the net mouth is aimed at the shrimp school. At this time, the pump-suction vessel conducts fishing at a low speed (the speed is greater than about 0.5 Kn and less than about 3 Kn, and the course change is large). The krill pump-suction vessel does not need to haul and set the trawl like a traditional trawler and can continuously fish for several days to thirty days. Excluding the time for detecting the shrimp school, it is in the fishing state for the rest of the time.

In this paper, the vessel position status of the vessel position points was identified based on the constructed CNN–attention model [19]. The CNN (Convolutional Neural Network), a deep learning model capable of automatically extracting spatial features from data, was integrated with an attention mechanism in this study’s CNN–attention model. This enabled the model to focus on critical features in vessel position data [19], such as speed and heading changes, thereby accurately determining fishing or non-fishing states and enhancing recognition accuracy. All AIS vessel position data points in the fishing time period of the fishing logs of the same type of Chinese Antarctic krill pump-suction vessels were marked as the fishing state, and the remaining data were marked as the non-fishing state. The five-dimensional data of binary classification (time, longitude, latitude, speed, course, status) were input into the model for training, and then the AIS vessel position data of the three Norwegian Antarctic krill pump-suction vessels were input into the model to determine the fishing and non-fishing states.

The duration of each state was calculated by taking the trajectory from the first vessel position point identified as each state every day to the next vessel position point identified as another state as the basis [20]. For example, the Antarctic krill pump-suction trawler usually sails at a high speed to the target fishing ground first, prepares for fishing, starts low-speed pump-suction fishing after aiming the pump-suction net mouth at the krill, and then sails at a high speed to the target fishing ground again.

The calculation formula for the duration of each state of the pump-suction trawl is shown in Formula (1):

(1) $$T=\sum_{i=1}^{n}\sum_{i=1}^{m}[P_{\left(j,i\right)}-P_{\left(j,i-1\right)}]$$

In Formula (1): *T* is the cumulative time (in hours, accurate to 0.1 h) of a certain state of a krill pump-suction trawler; *n* represents the number of trajectory segments; *j* represents the *j* trajectory segment, *m* is the number of vessel position points within this time period. The symbol *i* represents the *i*-th ship position point; *P*(*j*,*i*) − *P*(*j*,*i* − 1) is the time interval (in hours, accurate to 0.1 h) between two adjacent ship position points of the krill pump-suction trawler within a certain time period.

Due to the difference in spatial resolution between the ship position data and the environmental data, in order to ensure the matching of data resolution, before constructing the habitat model, it is necessary to re-grid the data. Therefore, in this paper, all the ship position data and environmental data are re-sampled to a spatial resolution of 0.083° × 0.083°, and the ship position data and environmental data are matched on a monthly time scale.

### 2.3. Habitat Simulation Based on the Integrated Species Distribution Model

The integrated species distribution model (ISDM), as a cutting-edge predictive technical method, constructs a comprehensive overall prediction framework by integrating multiple single species distribution models (SDMs). Among them, the single species distribution models involved cover various types such as ANN (Artificial Neural Network), CTA (Classification and Regression Tree Analysis), FDA (Flexible Discriminant Analysis), GAM (Generalized Additive Model), GBM (Gradient Boosting Machine), GLM (Generalized Linear Model), MARS (Multivariate Adaptive Regression Splines), and MAXNET (Maximum Entropy Neural Network). This method was proposed by Araújo et al. [21], and its core principle focuses on fusing the prediction results generated by multiple independent species distribution models. Compared with a single species distribution model, the integrated species distribution model can effectively reduce the internal bias and variance of the model, and thus significantly enhance the generalization performance of the entire model [5,22,23]. Based on these remarkable advantages, the integrated species distribution model has demonstrated excellent application effectiveness in the prediction of habitats of many marine species [24].

The relevant parameters of the algorithm are adjusted using RStudio 4.2.3. The fishing points identified based on the ship position data and the marine environmental data (SST, SSH, CHL, SIC, SSS, GLD) are input to predict the habitat of krill. The data are divided into a training set accounting for 80% and a test set accounting for 20%. Cross-validation is adopted during the model training and evaluation stage. The cross-validation strategy is set as randomly dividing the training set and the test set to ensure the randomness of each division, and the cross-validation process is repeated to more stably estimate the model performance. An integrated algorithm is used to construct the model. On a monthly basis, TSS is used as the screening index with a threshold set at 0.85. Several species distribution models with TSS values higher than 0.85 are screened out for combination, and a weighted average is used to construct the integrated distribution model for each month in the integrated algorithm [23].

The “bm_PlotResponseCurve” function in the biomod2 software package is used to generate environmental response curves for the integrated species distribution model [23]. The environmental response curves allow us to visualize and analyze the relationship between the six environmental variables and the probability of the presence of Antarctic krill, and to understand how each environmental variable affects the habitat suitability of the species and determine the optimal habitat conditions under these variables.

AUC and TSS are important evaluation indicators in the integrated species distribution model [25]. The AUC value represents the area under the ROC curve. The larger the area, the better the classification performance. The range of the AUC value is from 0 to 1, and the higher the value, the better the model performance [26]. TSS is a comprehensive evaluation indicator that comprehensively considers the difference between the model’s ability to correctly identify positive cases (true positive rate) and the ability to mistakenly identify negative cases as positive cases (true negative rate). The range of the TSS value is from −1 to 1, and the higher the value, the higher the accuracy [27].

### 2.4. The Relationship Among the Change in Antarctic Krill’s Habitat Area, Fishing Duration, and Catch

In the integrated species distribution model, the Habitat Suitable Index (HSI) represents the probability of the occurrence of a species, which is generated by combining the predictions of each model through a weighted average. In biomod2, the HSI ranges from 0 to 1000. Taking the month as the time scale, we used ArcGIS 10.6 software to draw the distribution map of the habitat of Antarctic krill. In order to more intuitively express the distribution of HSI, the HSI index is normalized to the range of 0–1, and according to the habitat index, it is divided into four suitable habitat areas: HSI ≥ 0.8 (highly suitable habitat area), 0.8 > HSI ≥ 0.6 (moderately suitable habitat area), 0.6 > HSI ≥ 0.4 (lowly suitable habitat area), 0.4 > HSI ≥ 0 (unsuitable habitat area) [23].

The distribution areas of these four suitable habitat areas are calculated, respectively. The distribution and area of the habitat of the krill fishing ground in each month are calculated through ArcGIS 10.6 software. The area calculation formula is:

(2) $$A=R^{2}\cos\left(\varphi \right)\times dx\times dy\times {\left(\frac{\pi }{180}\right)}^{2}$$

In Formula (2), A represents the area of a single grid (unit: km^2^); *R* is the average radius of the Earth (unit: km); φ is the latitude of the grid center; *dx* is the longitude range; *dy* is the latitude range.

(3) $$S_{K}=\sum_{C\left(r_{ij}\right)=k}A\left(r_{ij}\right)$$

In Formula (3), *S_K_* represents the total area of a certain adaptive region (unit: km^2^); *C*(*r_ij_*) *= k* is a certain pixel point in a certain adaptive region; *A*(*r_ij_*) is area of a certain pixel point.

The relationship between the area of each HSI region, the catch, and the fishing duration was analyzed through linear fitting based on the *t*-test [28] to explore the influence degree of habitat adaptability and fishing duration on the catch, as well as the potential driving factors of catch change. In RStudio, the *t*-test was used to check whether there was a significant difference (significant: *p* < 0.05; extremely significant: *p* < 0.01) [29].

Linear fitting based on the *t*-test:

(4) $$Y=\beta_{1}\times \beta_{0}+\varepsilon$$

In Formula (4), *Y* represents the catch; x represents different independent variables (area or duration); $\beta_{0}$ represents the intercept $\beta_{1}$ represents the slope; ε represents the error term.

In RStudio, the R-squared (*R*^2^) test is used to evaluate the linear fit. It measures the degree to which the model explains the variation of the dependent variable. The value of (*R*^2^) ranges from 0 to 1. The closer the value is to 1, the better the model’s fit and the stronger its ability to explain the variation of the dependent variable [30].

(5) $$R^{2}=\frac{\sum_{i=1}^{n}{\left(y_{i}-\hat{y_{i}}\right)}^{2}}{\sum_{i=1}^{n}{\left(y_{i}-\bar{y}\right)}^{2}}$$

In Formula (5), $R^{2}$ represents the coefficient of determination; n represents the number of data points involved in the linear fitting; $y_{i}$ represents the actual observed value of the dependent variable; $\hat{y_{i}}$ represents the value of the dependent variable predicted by the linear fitting; $\bar{y}$ represents the mean value of the dependent variable.

## 3. Results

### 3.1. Performance Evaluation of the Integrated Species Distribution Model

**Figure 2:**
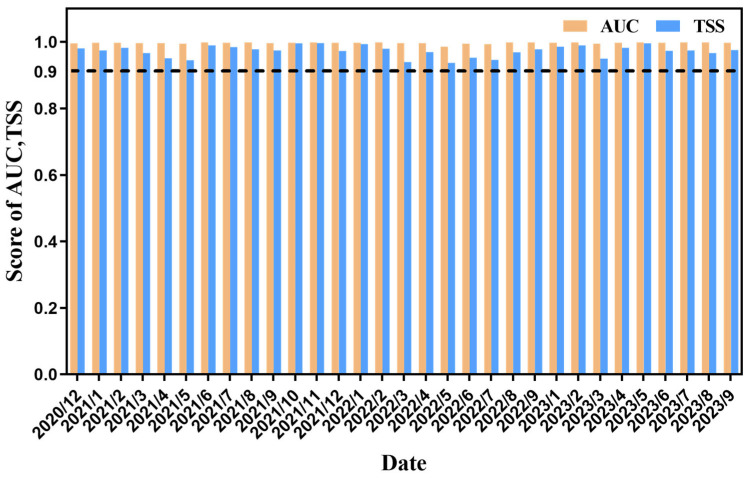
Performance evaluation of the integrated species distribution model of krill habitat for each month from 2020 to 2023.

The AUC (Area Under the Curve) values and TSS (True Skill Statistic) values of the integrated species distribution model for each month are all significantly greater than 0.9. Among them, the average AUC value is 0.997, and the average TSS value is 0.973. This indicates that the integrated species distribution model adopted in this study has demonstrated excellent performance and a high degree of reliability in terms of the prediction results (Figure 2).

### 3.2. Contribution Rates of Environmental Factors and Distribution of Krill Habitats

**Figure 3:**
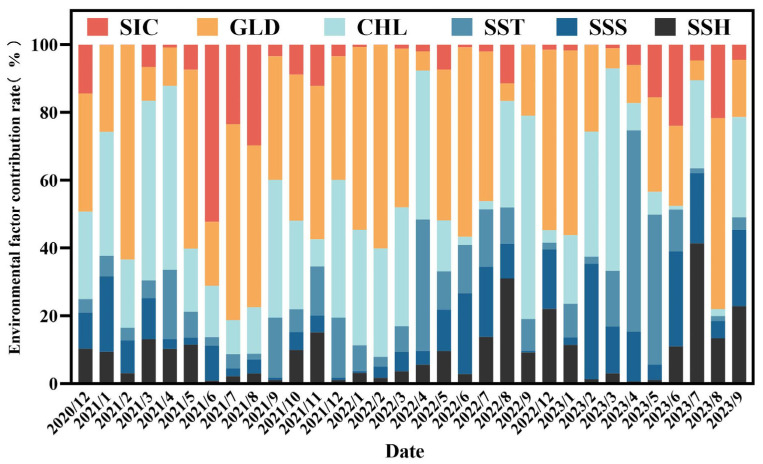
Contribution rates of environmental factors for each month.

The integrated species distribution model was employed to analyze the contributions of environmental factors. The results showed that among all environmental factors, the relative monthly average contribution rate of GLD ranked first, reaching 34.2%, and its contribution rate in each month demonstrated high stability (Figure 3). The relative monthly average contribution rate of CHL followed, at 25.8%, also remaining relatively stable across months. The cumulative monthly average contribution rate of GLD and CHL exceeded 60%, indicating that GLD and CHL are the key environmental factors playing a dominant role in the distribution of Antarctic krill habitats. The relative monthly average contribution rates of SSH and SST were around 10%, suggesting that they have a certain degree of influence on the distribution of Antarctic krill habitats. The relative monthly average contribution rate of SIC was approximately 7.6%, but from May to August, its monthly average contribution rate could reach 13.5%, showing that SIC has a relatively important influence on the distribution of Antarctic krill habitats during this period. The relative monthly average contribution rate of SSS was about 9.9%, with a relatively stable contribution rate across months. In summary, different environmental factors play unique and important roles in the distribution of Antarctic krill habitats through their varying contribution rates. These results provide crucial evidence for an in-depth understanding of the formation mechanism of Antarctic krill habitats and the influencing factors of the ecological environment.

**Figure 4:**
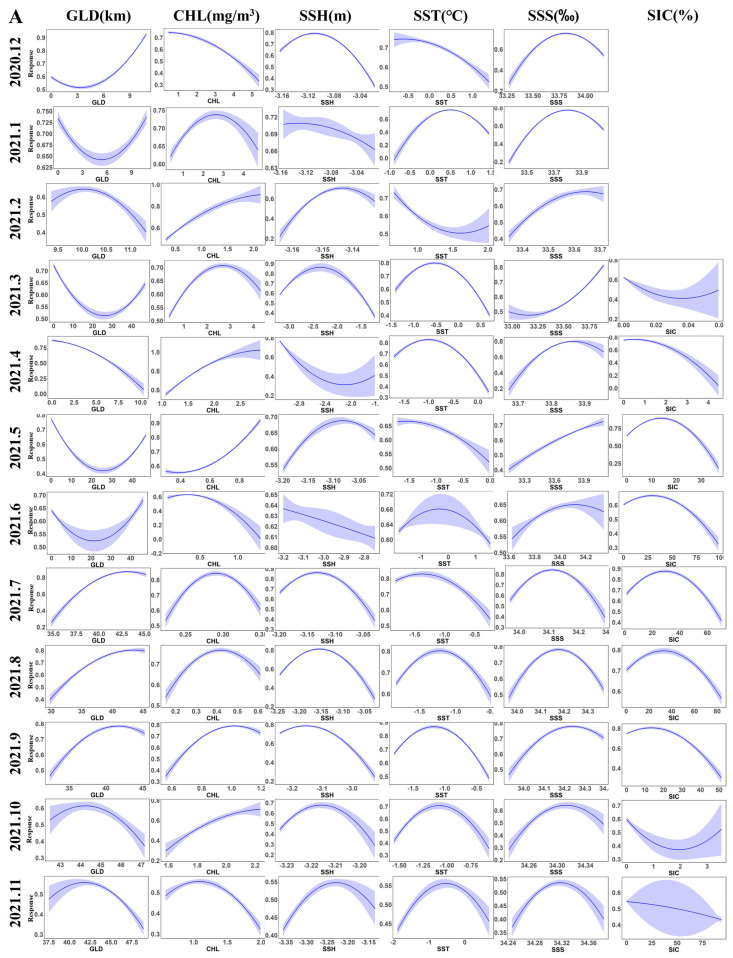

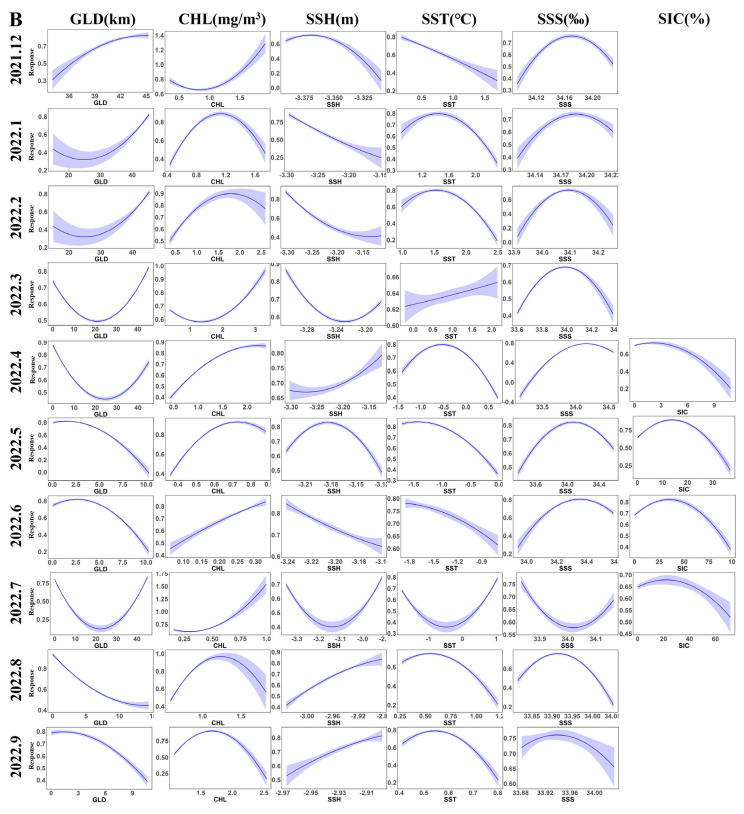

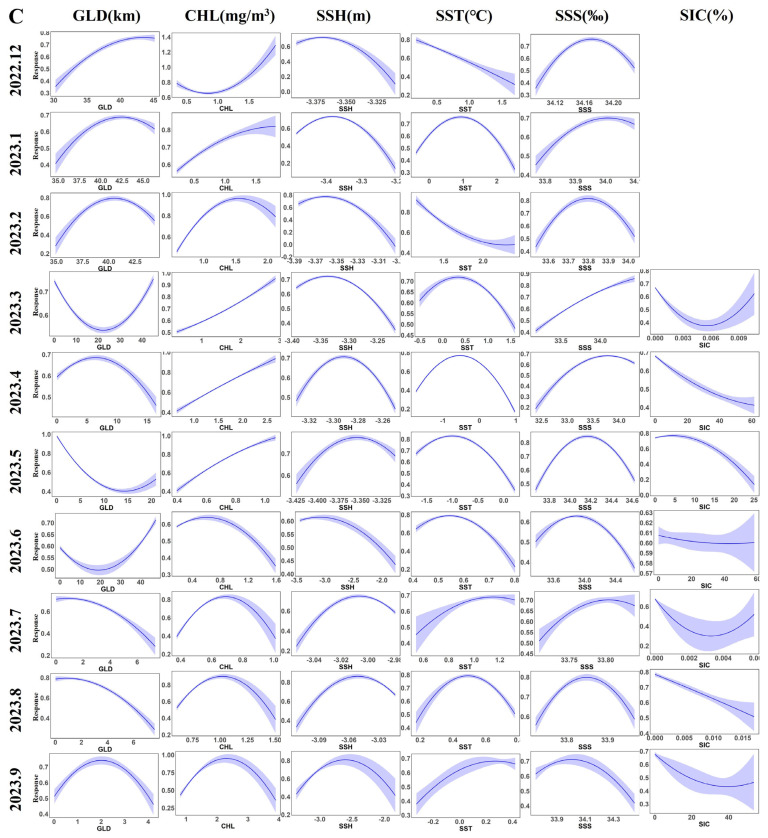
Response curves of various environmental factors for the habitats of krill resources ((**A**): fishing season in 2021; (**B**): fishing season in 2022; and (**C**): fishing season in 2023) (HSI ≥ 0.4).

Environmental response curves are effective tools for analyzing the complex relationships between individual environmental factors and habitat suitability. The environmental response curves for each month are presented in Figure 4A–C. The ranges of environmental factors corresponding to the highly suitable habitat areas (HSI > 0.8) each month were statistically analyzed, and the values of each environmental factor corresponding to the peak HSI each month were extracted. The relevant data are listed in Table 1. Among them, the GLD values vary according to different sub-areas. In Sub-areas 48.1 and 48.3, the GLD values are relatively low, while in Sub-area 48.2, the GLD values are relatively high. CHL shows characteristics of changing with the seasons, with higher values in summer and lower values in winter. SST and SIC are not only affected by seasonal changes but also related to differences in sub-areas. During operations in Sub-areas 48.1 and 48.2 in summer, their values are relatively high, and the situation is the opposite in winter. When operating in Sub-area 48.3 in winter, their values are relatively high. The values of SSH and SSS are relatively stable throughout the year with little fluctuation.

**Figure 5:**
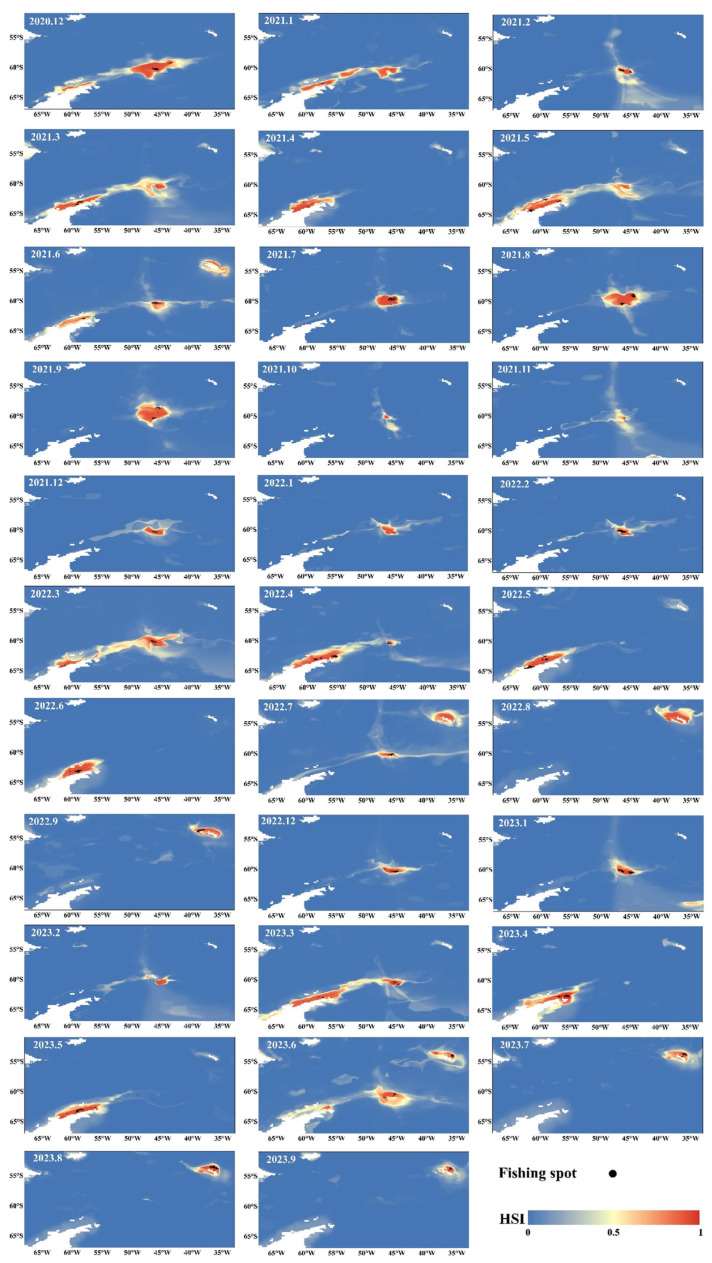
Distribution of habitat suitability index of krill resource habitats for each month.

The integrated species distribution model was used to analyze the habitats of Antarctic krill. The results indicated that the habitats of Antarctic krill are mainly concentrated in Sub-areas 48.1 and 48.2 in spring and summer (from September to February of the following year), and the main habitats gradually shift to Sub-areas 48.2 and 48.3 in autumn and winter (from March to August) (Figure 5).

### 3.3. The Relationship Among the Habitat Area of Krill, the Fishing Duration, and the Catch

**Figure 6:**
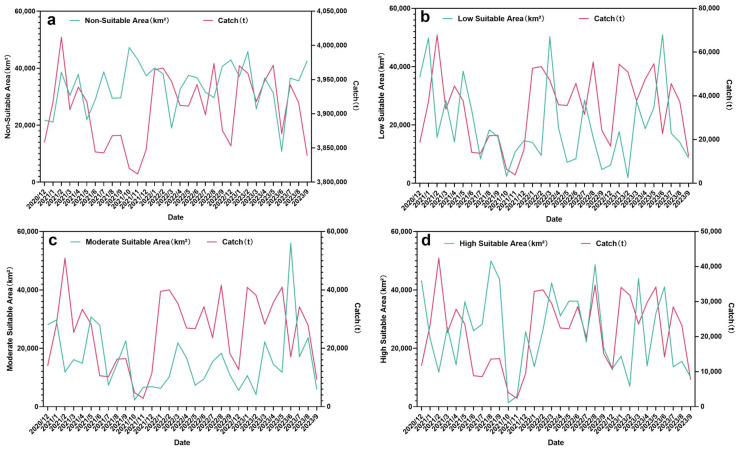
Changes in the area of each suitable habitat zone and the catch per month ((**a**): area of the unsuitable habitat zone; (**b**): area of the low suitable habitat zone; (**c**): area of the medium suitable habitat zone; and (**d**): area of the high suitable habitat zone).

When the operations of the three Norwegian fishing vessels are relatively scattered, the areas of the low, medium, and high suitable habitat zones are often relatively large (Figure 5 and Figure 6). From the perspective of the fishing seasons from 2021 to 2023, the peak value of the suitable habitat area gradually shifts from the early stage of the fishing season to the middle stage, and then to the later stage. In contrast, the change in the valley value of the area of the unsuitable habitat zone is opposite to that of the suitable habitat zone. The area of the suitable habitat is the smallest, and the area of the unsuitable habitat is the largest at the end of the fishing season. At the end of the fishing season, the catch decreases as the areas of the low, medium, and high suitable habitat zones shrink. At the end of the fishing season, in the unsuitable habitat zone, the catch decreases as the area increases. In addition, there is no obvious pattern between the area of each suitable habitat zone and the catch (Figure 6a–d).

**Figure 7:**
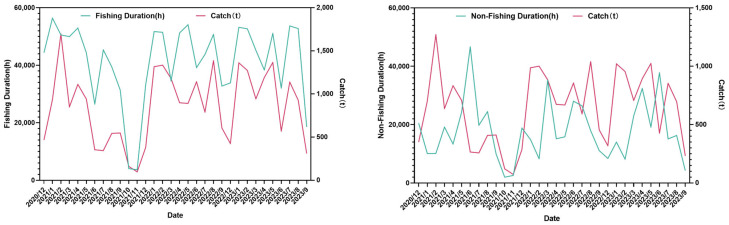
Changes in each fishing duration and the catch per month ((**a**): fishing duration; and (**b**): non-fishing duration).

The fishing duration and the non-fishing duration reach their valley values at the end of the fishing season, and there is no obvious pattern in other periods. The catch increases as the fishing duration increases. As for the relationship between the non-fishing duration and the catch, at the end of the fishing season, the catch also decreases as the non-fishing duration increases (Figure 7a,b).

**Figure 8:**
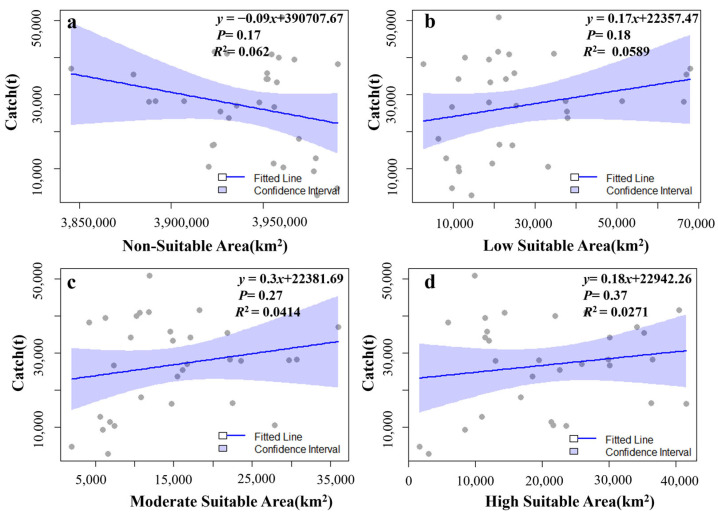
Linear fitting relationships between the area of each suitable habitat zone and the catch (**a**–**d**).

**Figure 9:**
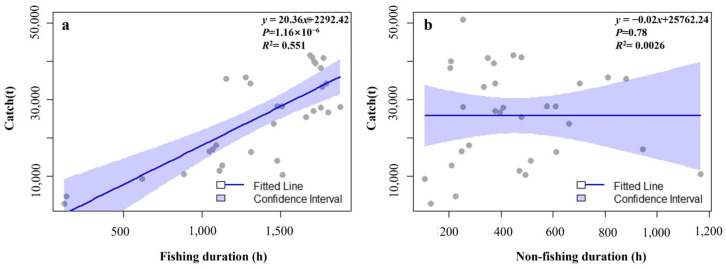
Linear fitting relationships between the fishing duration, non-fishing duration, and the catch (**a**,**b**).

In order to further explore the relationship among the area, the duration, and the catch, the area data were fitted with the monthly catches (Figure 8). Among them, there is a negative correlation between the area of the unsuitable habitat zone and the catch, with a *p* value of 0.17 and an *R*^2^ value of 0.062. There are positive correlations between the areas of the low, medium, and high suitable habitat zones and the catch, with *p* values of 0.18, 0.27, and 0.37, respectively, and *R*^2^ values of 0.0589, 0.0411, and 0.0271, respectively. The operation durations of the Norwegian pumping vessels each month were calculated and fitted with the catches (Figure 9). It was found that there is a significant positive correlation between the fishing duration and the catch, with a *p* value of 1.16 × 10^−6^ and an *R*^2^ value of 0.551. There is a negative correlation between the non-fishing duration and the catch, with a *p* value of 0.78 and an *R*^2^ value of 0.0026. Except for the significant relationship between the fishing duration and the catch, none of the other variables shows a good correlation.

## 4. Discussion

### 4.1. Accuracy of the Integrated Species Distribution Model Based on Vessel Position Data

Antarctic krill is an important economic species. A small number of scholars have conducted many studies on the habitats of Antarctic krill. These studies mainly use data such as collected fishing logs and on-site scientific surveys combined with environmental factors to describe the habitats of Antarctic krill [2,5,31,32,33]. There is no analysis of the habitats of Antarctic krill based on vessel position data. Therefore, compared with previous studies, this study is based on vessel position data, extracts the fishing ground positions when the vessels are in the fishing state, and sets them as the predictive variables of the integrated species distribution model. The reason for adopting this approach is that, compared with traditional fishery data, the fishing state of the vessels extracted from the vessel position data can more truly and effectively reflect the behavioral characteristics of the vessels during the actual fishing process. It should be clear that the core purpose of Antarctic krill fishing vessels entering the fishing area for operation is to obtain catches. Every period of time that a vessel stays at sea will inevitably increase the corresponding costs and may even incur additional costs and expenditures [34]. Therefore, analyzing and mastering the spatio-temporal distribution characteristics of the optimal habitats of Antarctic krill can provide guidance for fishing activities to a certain extent. In this study, the vessel positions of Norwegian pumping vessels were selected as the research objects because Norway started early in the field of krill fishing and has mastered advanced fishing technologies, and its krill fishery level is at the world’s leading position [35,36]. Therefore, in-depth exploration of the fishing habitats of Norwegian krill vessels is of great guiding significance for global krill fishing.

Due to the difficulty of obtaining foreign fishing logs, and the lengths, tonnages, and fishing methods of pumping vessels at home and abroad are relatively similar, this study uses Chinese fishing logs as a basis to classify the status of the vessel position data of Chinese pumping vessels of the same type, and it is highly credible to estimate the fishing behavior of Norwegian pumping vessels. Subsequently, we input the vessel position data after status classification into the constructed deep learning model for training. Through this process, it is possible to achieve the status recognition of the vessel position data of fishing vessels of the same type.

In this study, the vessel position data of Antarctic krill vessels and the integrated species distribution model were used to construct their habitats. The AUC and TSS indices of the model for each month exceed 0.9, with an average AUC of 0.997 and an average TSS of 0.973. It can accurately define the distribution of habitats, monitor changes, and identify fishing hotspots. Although vessel position data have significant advantages in terms of real-time performance and spatial coverage [37], vessel position data have certain limitations. Vessel position data can only reflect the operation points of fishing vessels and cannot provide information on the catch of each haul, which may lead to false positive or false negative results in habitat prediction. However, such errors are extremely rare for Antarctic krill fishing because the resource density of Antarctic krill is relatively high, and in actual fishing, it is very rare to have an empty net or a low catch (less than 5 tons per net).

### 4.2. Key Environmental Factors of the Habitat of Krill and Their Impacts

Antarctic krill play a pivotal role in the material cycling and energy flow of the Antarctic ecosystem. In this study, an integrated species distribution model was employed to analyze the impacts of various environmental factors and quantify the relative contributions of each factor to the distribution of krill habitats, thus providing a scientific basis for revealing the driving mechanisms of the habitats.

Since krill fishing vessels operate in different sub-regions in some months, it is likely to cause errors in the suitable ranges of environmental factors. Therefore, the suitable ranges of environmental factors corresponding to the highly suitable habitats (HSI ≥ 0.8) when operating solely in a specific sub-region were selected for discussion. As shown in Figure 3 and Figure 4 and Table 1, GLD, as a key determinant of basicity and stability, plays an extremely important role in habitat suitability, with an average monthly contribution rate of approximately 34.9%. Moreover, the environmental response curves of GLD exhibit significant differences in different operating sub-regions. In sub-regions 48.1 and 48.3, the GLD values are at a relatively low level (GLD < 12 km), and the peak suitable range is 0.35–2.45 km, while the GLD values in sub-region 48.2 are relatively high (30–50 km), with a peak suitable range of 43.05–44.66 km. This phenomenon may be due to the fact that during the seasonal alternation, the distribution of krill will migrate back and forth according to the extension direction of the continental shelf [38,39], or it is related to the fact that krill inhabit the ice edge zone and their distribution is affected by the expansion and retreat of sea ice. Some studies have shown that within the range of 0–50 km, the biomass of krill groups is more than twice that in waters beyond 200 km [40].

CHL is directly related to the food supply of krill. In spring and summer, the massive proliferation of phytoplankton is a key factor affecting the recruitment of krill populations near the Antarctic Peninsula, with an average monthly contribution rate of approximately 25.2%. When it is spring and summer (from September to February of the following year) and the CHL concentration is relatively high, the suitable range is 0.34–2.68 mg/m^3^, and the peak suitable range is 1–2.13 mg/m^3^. At this time, the environmental factor response curve of CHL shows a trend of rising first and then falling. It is worth noting that when choosing habitats, krill do not solely rely on areas with high CHL concentrations, because high-resource areas are often accompanied by more predators, resulting in a higher predation risk for krill. Therefore, krill may choose to inhabit areas with relatively less food resources but lower predation risks to balance resource acquisition and their own survival security, thereby ensuring the stability of the population [38]. In autumn and winter (from March to August), the CHL concentration is relatively low, with a suitable range of 0.19–2.6 mg/m^3^ and a peak suitable range of 0.27–2.05 mg/m^3^, and the corresponding environmental factor response curve shows an upward trend. This may be because food is relatively scarce in winter, and krill will tend to choose areas with relatively higher CHL concentrations for foraging activities in order to obtain sufficient food. Some studies have shown that the distribution of CHL in the Southern Ocean exhibits obvious spatiotemporal distribution characteristics, with higher CHL values near islands and in shelf waters [41], and the high aggregation areas of Antarctic krill are basically located in the inshore waters with relatively higher CHL concentrations [2,42]. In this study, it was found that GLD and CHL interact with each other, and their comprehensive contribution rate exceeds 60%, jointly determining the habitat suitability and constituting the core environmental framework of habitat suitability, thus further confirming the above conclusions.

The average monthly contribution rates of SSH and SST are both approximately 10%. Although their influence is relatively limited, they can change the marine hydrological conditions, thereby affecting the distribution of phytoplankton and the physico-logical adaptability of krill and further regulating the habitat suitability. SSH mainly affects the distribution of phytoplankton by changing the ocean currents and water body structure. Its annual variation range is small, with an adaptation range of −3.43 to −2.9 m, a peak suitable range of −3.41 to −2.91 m, and the environmental response curve shows a trend of rising first and then falling. SSH is related to the water depth. The hydrological environment in deep waters is complex, and krill have weak swimming abilities and are easily affected by hydrological dynamic factors such as ocean currents and eddies. For example, mesoscale eddies may become suitable habitats for krill due to their high primary productivity [6]. Small fluctuations in SST can have a significant impact on the physiological status of Antarctic krill. The suitable SST varies in different sub-regions. The suitable SST in sub-region 48.1 is −1.78 to 0.73 °C, with a peak suitable range of −1.75 to −0.54 °C; the suitable SST in sub-region 48.2 is −1.81 to 1.98 °C, with a peak suitable range of −1.61 to 1.4 °C; the suitable SST in sub-region 48.3 is −0.18 to 1.29 °C, with a peak suitable range of 0.34 to 1.03 °C. Under low temperature conditions, the probability of species occurrence shows an upward trend, while it shows a downward trend at relatively high temperatures. The suitable SST in each sub-region is consistent with the research results of Zhu Guoping [43], but the peak range is smaller than that in Zhu Guoping’s research. This may be due to the natural fluctuations of the marine environment between different years and the differences in the operation modes of the vessels.

SIC shows an obvious seasonal influence, with an average monthly contribution rate usually only 7.6%, but in late autumn and winter (from May to August), this contribution rate will increase to 13.5%, with a suitable range of 0–75.92% and a peak suitable range of 0–27.73%. Its dynamic changes not only contribute to the growth of phytoplankton but also provide a sheltering effect for krill habitats, especially during the critical stages of krill reproduction and growth. Its environmental response curve shows an upward trend when SIC is at a relatively high level, which is consistent with the research results of Chen Feng [44] and others. However, as SIC continues to rise, the environmental response curve shows a downward trend. This may be because SIC poses a danger to ships, causing them to actively stay away from areas with high SIC density for fishing operations, thus leading to a decrease in environmental adaptability [45]. SSS plays a key role in providing long-term guarantees for the physiological metabolism and reproductive activities of krill, with an average monthly contribution rate of up to 9.9%. It is worth noting that the differences in SSS in different seasons and different sub-regions are relatively small, with a suitable range of 33.61–34.69‰ and a peak suitable range of 33.82–34.29‰, and the corresponding environmental response curve shows a trend of rising first and then falling, which is consistent with the research results of Zhao Guoqing and others [8]. Ship position factors and policy factors also have a certain impact on the habitat selection and distribution patterns. Fishing vessels usually avoid entering quota areas or areas with high SIC density due to fishing quotas or to avoid the dangers posed by SIC, thus affecting the development and utilization of habitats. In addition, this article further explored the roles of these environmental factors in the formation of Antarctic krill habitats and their seasonal migration processes through a detailed analysis of sub-regions 48.1, 48.2, and 48.3. In sub-region 48.1, the seawater temperature is suitable, and phytoplankton are abundant in summer. The warm seawater promotes the growth of phytoplankton [46], the habitat suitability is high, and the food is sufficient. However, in winter, the SST decreases, the habitat suitability drops significantly [47], the area of krill habitats shrinks, and krill migrate to sub-region 48.2. Its habitat is sensitive to temperature and is closely related to the fluctuations of GLD [48]. Affected by the expansion of SIC and fishing quotas, the habitat distribution shows distinct seasonality and depends on a warm and stable environment. The habitat conditions in sub-region 48.2 are stable throughout the year. The GLD value is high, and the living space is vast. The high CHL in spring and summer (from September to February of the following year) is conducive to the growth and reproduction of krill. In autumn and winter (from March to August), the increase in SIC has little impact on the suitability. The environment is stable during the seasonal alternation, and the suitability is good throughout the year. The habitat suitability in sub-region 48.3 shows obvious seasonal fluctuations [43]. In winter, the increase in SIC concentration reduces the habitat area in other sub-regions, and krill concentrate in deep waters [49]. Although the overall area of this region is small, due to SIC and fishing quotas in winter, fishing vessels avoid it, making it a relatively stable winter fishing operation area.

In conclusion, the distribution and seasonal changes in Antarctic krill habitats are the result of the collaborative effects of multiple environmental factors.

### 4.3. The Relationship Between the Habitat Area of Antarctic Krill, the Fishing Duration, and the Catch

It is generally believed that high-quality habitat has a positive promoting effect on the inhabitation and reproductive activities of fishery populations. Specifically, there is a close correlation between the area of high-quality habitat and the abundance of resources. Generally speaking, the larger the area of high-quality habitat, the higher the abundance of resources tends to be; conversely, if the area of high-quality habitat decreases, the abundance of resources will decrease accordingly [47]. And fishing operations carried out in areas with high resource abundance usually can catch higher fishing production. As a kind of large zooplankton, krill has obvious clustering characteristics [50] and a relatively high resource density [48]. Its resource distribution and fishing production will inevitably be affected by the suitable habitat areas of different habitats. However, there is a severe lack of relevant research on this impact effect at present.

This study found that there are different relationships between the area of different suitability regions and production. By fitting the area of the unsuitable habitat zone and the production, it was found that there is a negative correlation between them. From the perspective of the *p* value and *R*^2^, there is no significant relationship, and the fitting relationship is poor. But there is a probability that when the area of the unsuitable habitat zone increases, the production decreases. And at the end of the fishing season, the area of the unsuitable habitat zone increases significantly (Figure 6a), because the vessels gradually leave the fishing ground, resulting in a decrease in the area of the suitable habitat zone. The fitting of the areas of the low, medium, and high suitable habitat zones to the production shows a positive correlation. However, the *p* value and *R*^2^ also perform poorly. There is a probability that when the area increases, the production also increases. At the end of the fishing season, the area of the suitable habitat zone decreases significantly (Figure 6b,c). These conclusions indicate that although the habitat area can reflect the level of krill production indirectly, it cannot accurately predict the specific fishing volume level. It reflects that it is still very difficult to quantify the future production level of krill based on the fishing effort of the habitat area.

In contrast, there is a significant positive correlation between the fishing duration and the production. The *p* value and *R*^2^ perform well, indicating that the fishing duration is an important factor affecting the fishing production. The extension of fishing operation time can significantly increase the production [51]. A longer fishing operation time means that the fishing vessels increase the probability of encountering krill groups, and at the same time, they can obtain more catches during the period when krill are concentrated, which is intuitively reflected in the final fishing production data [34]. This reflects that the continuity and input degree of fishing activities have a direct impact on the fishing of krill resources [52]. There is a negative correlation between the non-fishing duration and the production, but both the *p* value and *R*^2^ perform poorly, and the relationship with the production is not significant. In general, the significant positive correlation between the fishing duration and the production indicates that extending the fishing operation time is an effective way to increase the krill production [50]. Therefore, future krill fishing management can focus on the optimization of the fishing duration, and then scientifically and reasonably plan the time arrangement of fishing operations to ensure that fishing vessels can efficiently go to areas with high resource density for fishing activities within a given and relatively limited time range. In this way, not only can the fishing efficiency be effectively improved, so that the catch per unit time can be significantly increased, but also it is helpful to improve the economy of fishing operations, ensure the sustainable development of the krill fishing industry, and achieve a virtuous balance between ecological benefits and economic benefits.

## 5. Conclusions

Vessel position data (AIS) and the species distribution model (ISDM) demonstrated significant practical value in constructing species distribution models under data-limited conditions. The research results show that: (1) Offshore distance and chlorophyll concentration are important environmental indicators affecting Antarctic krill habitat; (2) There is no significant correlation between habitat area and catch; (3) Fishing operation duration exhibits a strong positive correlation with catch. Performance metrics (AUC, TSS) of the model indicate that the constructed integrated species distribution model demonstrates excellent performance and high reliability in prediction results.

When analyzing Antarctic krill habitats, vessel position data outperforms traditional commercial fishing logs in terms of accessibility, data volume, accuracy, and spatiotemporal continuity. This enables the model to capture the spatiotemporal distribution characteristics of Antarctic krill when fitting fishing locations more precisely with environmental variables. Although vessel position data have obvious advantages over traditional commercial fishing logs, they can only characterize the operational position features of fishing vessels and lack biomass information, such as per trawl catch, which may lead to misjudgment risks in habitat prediction. Future research can integrate fishing gear specifications, pumping efficiency, and other operational characteristics data to construct catch estimation models, thereby further improving the model.

## Figures and Tables

**Table 1:** Ranges of environmental factors corresponding to highly suitable habitat areas each month and peak values of environmental response curves (values in parentheses) (HSI ≥ 0.8).

Month	GLD (km)	CHL (mg/m^3^)	SSH (m)	SST (℃)	SSS (‰)	SIC(%)
2020.12	0.84~44.93 (43.24)	0.59~3.83 (0.83)	−3.15~−3.05 (−3.13)	−0.59~1.12 (0.39)	33.51~34.16 (33.88)	0~0.01 (0)
2021.01	0.57~44.85 (2.48)	0.57~3.77 (2.91)	−3.14~−3.03 (−3.04)	0.04~1.05 (0.48)	33.54~33.99 (33.84)	0~0 (0)
2021.02	2.29~2.54 (2.4)	0.45~2.13 (1.87)	−3.16~−3.14 (−3.15)	0.67~1.32 (0.83)	33.44~33.65 (33.53)	0~0 (0)
2021.03	0.21~44.17 (1.34)	0.57~3.73 (3.06)	−3.18~−3.04 (−3.06)	−0.51~0.87 (−0.16)	33.48~33.89 (33.81)	0~0 (0)
2021.04	0.04~4.86 (0.76)	1.21~2.38 (2.05)	−3.1~−3.06 (−3.07)	−1.28~0.37 (−1.08)	33.76~33.88 (33.83)	0.01~1.4 (0.38)
2021.05	0.21~44.37 (1.18)	0.36~0.94 (0.85)	−3.19~−3.03 (−3.04)	−1.64~−0.54 (−1.53)	33.64~34.06 (33.83)	0.16~15.89 (4.64)
2021.06	1.26~45.48 (44.25)	0.24~0.42 (0.29)	−3.18~−2.76 (−3.18)	−1.81~1.36 (−1.63)	33.79~34.19 (33.85)	0~67.69 (12.13)
2021.07	38.04~44.93 (43.96)	0.23~0.33 (0.3)	−3.19~−3.06 (−3.11)	−1.81~−0.34 (−0.73)	33.98~34.22 (34.1)	3.28~58.06 (7.44)
2021.08	33.85~44.93 (44.12)	0.19~0.6 (0.42)	−3.23~−3.07 (−3.19)	−1.79~−0.57 (−1.61)	34.02~34.29 (34.21)	1.53~75.92 (27.73)
2021.09	35.24~44.85 (44.46)	0.7~1.13 (1.06)	−3.24~−3.08 (−3.21)	−1.77~−0.49 (−1.58)	34.05~34.39 (34.26)	0~36.37 (5.04)
2021.10	43.75~44.85 (44.66)	1.9~2.15 (2.13)	−3.22~−3.22 (−3.22)	−1.27~−1.07 (−1.21)	34.29~34.31 (34.3)	0~0 (0)
2021.11	42.35~44.3 (43.32)	0.81~1.18 (1.04)	−3.24~−3.23 (−3.24)	−0.37~−0.33 (−0.35)	34.29~34.31 (34.29)	0~0 (0)
2021.12	37.97~44.93 (43.68)	0.55~1.16 (1)	−3.27~−3.25 (−3.26)	0.48~0.98 (0.59)	34.23~34.33 (34.3)	0~0 (0)
2022.01	39.84~45.16 (44.65)	0.63~1.74 (1.18)	−3.29~−3.26 (−3.27)	1.15~1.97 (1.23)	34.15~34.22 (34.19)	0~0 (0)
2022.02	39.84~45.16 (44.65)	0.53~2.68 (1.42)	−3.3~−3.26 (−3.28)	1.3~1.98 (1.31)	34.02~34.11 (34.09)	0~0 (0)
2022.03	0.04~45.26 (44.06)	0.51~3.31 (1.69)	−3.3~−3.19 (−3.29)	−0.22~1.9 (1)	33.94~34.06 (34.04)	0~0.01 (0)
2022.04	0.04~44.93 (1.4)	0.59~2.38 (1.69)	−3.29~−3.15 (−3.21)	−1.23~0.38 (−0.77)	33.8~34.49 (34.3)	0.01~4.66 (0.35)
2022.05	0.04~4.94 (0.43)	0.44~0.88 (0.76)	−3.23~−3.14 (−3.16)	−1.68~−0.26 (−1.23)	33.95~34.51 (34.08)	0.36~30.27 (9.46)
2022.06	0.26~5.88 (1.63)	0.17~0.33 (0.27)	−3.24~−3.17 (−3.21)	−1.78~−0.73 (−1.75)	34.17~34.56 (34.3)	0.14~34.83 (24.17)
2022.07	0.05~44.85 (0.22)	0.21~0.82 (0.65)	−3.34~−2.92 (−2.92)	−1.72~0.97 (0.71)	33.85~34.14 (33.88)	0~52.68 (0)
2022.08	0.01~6.71 (1.77)	0.66~1.53 (0.8)	−2.96~−2.92 (−2.93)	0.37~0.76 (0.59)	33.83~33.96 (33.88)	0~0 (0)
2022.09	0.05~6.34 (2.35)	1.19~1.82 (1.26)	−2.95~−2.9 (−2.91)	0.43~0.62 (0.58)	33.89~33.99 (33.9)	0~0 (0)
2022.12	36.81~44.99 (43.11)	0.36~1.92 (1.16)	−3.39~−3.36 (−3.37)	0.21~0.87 (0.39)	34.13~34.23 (34.18)	0~0 (0)
2023.01	39.04~45.16 (43.28)	0.34~1.82 (1.82)	−3.38~−3.36 (−3.37)	0.94~1.64 (0.99)	33.84~34.09 (34)	0~0 (0)
2023.02	37.83~43.92 (43.05)	0.7~2.12 (1.35)	−3.38~−3.35 (−3.36)	1.18~1.45 (1.4)	33.68~33.98 (33.69)	0~0 (0)
2023.03	0.04~44.67 (42.59)	0.4~2.75 (1.88)	−3.39~−3.29 (−3.39)	−0.14~1.22 (1.12)	33.45~34.42 (33.74)	0~0 (0)
2023.04	0.45~11.64 (2.45)	0.93~2.64 (2.16)	−3.32~−3.26 (−3.29)	−0.98~0.02 (−0.54)	33.61~34.27 (33.82)	0.02~2.54 (0.11)
2023.05	0.04~7.56 (0.35)	0.55~1.07 (0.85)	−3.41~−3.32 (−3.34)	−1.59~−0.5 (−1.43)	33.75~34.62 (34.15)	0.07~7.74 (2.58)
2023.06	0.05~45.26 (44.85)	0.22~1.19 (0.25)	−3.43~−2.98 (−3.41)	−1.74~2.11 (−0.94)	33.75~34.14 (33.79)	0~48.99 (8.56)
2023.07	0.05~3.81 (2.04)	0.47~1.01 (0.51)	−3.02~−2.98 (−3)	0.67~1.29 (1.03)	33.75~33.82 (33.8)	0~0 (0)
2023.08	0.05~3.71 (1.11)	0.68~1.51 (0.77)	−3.08~−3.02 (−3.03)	0.18~0.71 (0.58)	33.72~33.94 (33.83)	0~0.01 (0)
2023.09	0.05~3.38 (2.06)	1.05~1.99 (1.36)	−3.08~−3.03 (−3.04)	0.25~0.37 (0.34)	33.83~33.95 (33.88)	0~0 (0)

## Data Availability

The marine environmental data used in this study can be downloaded from the following three websites. https://gmed.auckland.ac.nz/download.html (accessed on 22 May 2025), https://data.marine.copernicus.eu/ (accessed on 22 May 2025).
